# Population Prevalence, Penetrance, and Mortality for Genetically Confirmed MODY

**DOI:** 10.1210/clinem/dgaf599

**Published:** 2025-11-01

**Authors:** Luke N Sharp, Kevin Colclough, Jacques Murray Leech, Stuart J Cannon, Thomas W Laver, Andrew T Hattersley, Michael N Weedon, Kashyap A Patel

**Affiliations:** Department of Clinical and Biomedical Sciences, Faculty of Health and Life Sciences, University of Exeter, Exeter EX4 4QJ, UK; Department of Clinical and Biomedical Sciences, Faculty of Health and Life Sciences, University of Exeter, Exeter EX4 4QJ, UK; Exeter Genomic Laboratory, Royal Devon University Healthcare NHS Foundation Trust, Exeter, UK; Department of Clinical and Biomedical Sciences, Faculty of Health and Life Sciences, University of Exeter, Exeter EX4 4QJ, UK; Department of Clinical and Biomedical Sciences, Faculty of Health and Life Sciences, University of Exeter, Exeter EX4 4QJ, UK; Department of Clinical and Biomedical Sciences, Faculty of Health and Life Sciences, University of Exeter, Exeter EX4 4QJ, UK; Department of Clinical and Biomedical Sciences, Faculty of Health and Life Sciences, University of Exeter, Exeter EX4 4QJ, UK; Department of Clinical and Biomedical Sciences, Faculty of Health and Life Sciences, University of Exeter, Exeter EX4 4QJ, UK; Department of Clinical and Biomedical Sciences, Faculty of Health and Life Sciences, University of Exeter, Exeter EX4 4QJ, UK

**Keywords:** MODY, diabetes, genetics, monogenic diabetes, penetrance

## Abstract

**Context:**

Diagnosing maturity-onset diabetes of the young (MODY) is clinically important for treatment and prognosis. However, phenotype-based studies of MODY are prone to ascertainment bias, limiting accurate estimates of its population prevalence and phenotypic spectrum.

**Objective:**

To apply a genotype-first approach to determine the population prevalence, penetrance, and all-cause mortality associated with MODY.

**Methods:**

We analyzed exome sequencing and clinical data from 454 275 UK Biobank participants to identify pathogenic variants in 10 established MODY genes. We assessed variant prevalence, age-dependent diabetes penetrance, and all-cause mortality by genetic etiology over a mean follow-up of 13.4 years.

**Results:**

Pathogenic MODY variants were present in 1 in 1052 individuals and accounted for 1.48% of diabetes cases diagnosed before age 40. *GCK* variants were the most frequent (1 in 2787), demonstrating high penetrance (mean HbA1c 8.8 mmol/mol higher; 94.5% with prediabetes or diabetes) but no significant association with all-cause mortality (*P* = .09). Variants in other MODY genes showed lower penetrance, with 12% of carriers developing diabetes by age 40 and 31.6% by age 60 and showed no increase in all-cause mortality (*P* = .89). Penetrance varied by genetic etiology, with *HNF1A* showing the highest penetrance and *PDX1*, *NEUROD1*, and *RFX6* the lowest. Parental history of diabetes and polygenic risk for type 2 diabetes were important modifiers of penetrance (hazard ratios 2.54 and 1.52, respectively; *P* < 3.9 × 10^−3^).

**Conclusion:**

This large-scale genotype-first study provides novel insights into MODY in the population. These findings have broad implications for genetic counseling, personalized treatment strategies, and healthcare resource allocation.

Our current understanding of maturity-onset diabetes of the young (MODY), particularly its prevalence, penetrance, and impact on mortality, has largely been shaped by studies using a phenotype-first approach. MODY is an autosomal dominant form of monogenic diabetes caused by pathogenic variants in at least 11 genes (1, 2). Accurate diagnosis is clinically important, as it directly informs treatment decisions and prognosis (1, 3, 4). Most studies on MODY have focused on young-onset diabetes that lack biomarkers for type 1 diabetes or familial diabetes identified from the routine clinical practice which are later confirmed to have genetic diagnosis of MODY (5-9). These studies are referred to as phenotype-first studies. Due to this reliance on phenotype selection, studies of monogenic disorders have been shown to suffer from ascertainment bias (5-11). This may lead to overestimation of age-dependent penetrance by focusing on individuals who develop diabetes at a young age (10). Similarly, due to homogenous selection criteria, these studies are potentially less powerful to assess the difference in phenotype due to underlying genetic etiology. The ascertainment bias of phenotype-first studies may also potentially explain the predominance of female cases in genetically confirmed MODY (4, 8, 12).

In contrast, genotype-first studies, which identify individuals based on genetic variants irrespective of phenotype, offer a powerful way to overcome the limitations of phenotype-first studies (11, 13). The lack of selection of the phenotype means they are well-suited to assessing the full clinical spectrum, prevalence, and outcomes, such as all-cause mortality, which remains unknown for most MODY subtypes. This is particularly relevant for *GCK*-MODY, which has a unique phenotype of lifelong mild fasting hyperglycemia, the effect of which on mortality has not been established. The previous genotype-first studies in MODY have been limited by restricted gene coverage (10), lack of mortality data (10, 14, 15), lack of age-dependent diabetes risk (14, 15), or reliance on case-control designs (15) and lack the assessment of factors affecting penetrance of diabetes (10, 14, 15), constraining their ability to provide comprehensive, population-level insights.

In this study, we leverage a genotype-first approach in a large population cohort from the UK to provide novel insights into MODY in the population. We aim to investigate the prevalence, age-dependent penetrance of diabetes, factors affecting penetrance, and all-cause mortality associated with MODY in a cohort of 454 275 individuals from the UK population.

## Methods

### Study Population

To assess the prevalence, penetrance, and all-cause mortality in the population, we used data from the UK Biobank, a large population-based study in the United Kingdom with participants aged between 40 and 70 years at recruitment (16). These participants provided detailed phenotypic information through electronic health records, self-reported questionnaires, and structured interviews. The UK Biobank study also measured biomarkers, including key diabetes-related markers such as HbA1c and blood glucose. Importantly, all participants also had comprehensive genetic data available. For this study, we analyzed whole exome sequencing from the 450 000-participant data release from the UK Biobank (16, 17). Table S1 provides the characteristics of the individuals included in the current study (18). Ethics approval for the UK Biobank study was obtained from the North West Centre for Research Ethics Committee (11/NW/0382). Written informed consent was obtained from all participants.

### Diabetes and All-Cause Mortality

We defined individuals with diabetes if they self-reported to have diabetes, were on diabetes medication, or had an HbA1c level >48 mmol/mol (6.5%) at the time of recruitment into the study. We incorporated self-reported age of diabetes diagnosis and the first occurrence data into our analysis. We defined prediabetes based on the American Diabetes Association (ADA) criteria, using a fasting glucose level ≥5.6 mmol/L or an HbA1c level ≥39 mmol/mol (19). We obtained all-cause mortality data up to November 2022 through the National Death Registries Linkage, which the UK Biobank carried out centrally. Detailed information is available at: https://biobank.ndph.ox.ac.uk/ukb/refer.cgi?id=115559.

### Genetic Data

We utilized the 454 275 participant whole exome sequencing release from the UK Biobank. Szustakowski et al described the detailed methodology for this sequencing, which is available at https://biobank.ctsu.ox.ac.uk/showcase/label.cgi?id=170 (17). Briefly, the IDT xGen Exome Research Panel v1.0 was used, which covered the exons of 19 396 genes. Samples were sequenced using the Illumina NovaSeq 6000 platform with mean coverage exceeding 20× at 95.2% of sites. Variants were called to the GRCh38 genome build reference sequence using the OQFE protocol (17).

We generated the type 1 diabetes genetic risk score (T1DGRS) and type 2 diabetes polygenic risk score (T2DPRS) using TOPMed imputed data genotyping array data released by the UK Biobank. Detailed information on this is available at https://biobank.ndph.ox.ac.uk/ukb/field.cgi?id=21007 and described by Arni et al (20, 21). We used a genome-wide T2DPRS developed by Huerta-Chagoya et al, which was derived from individuals excluding UK Biobank participants (22). To capture type 1 diabetes polygenic risk, we employed the T1DGRS2 developed by Sharp et al (20, 22, 23).

### Classification of MODY Variants

We analyzed variants in the coding regions of 10 genes known to cause MODY: *ABCC8*, *GCK*, *HNF1A*, *HNF1B*, *HNF4A*, *INS*, *NEUROD1*, *PDX1*, *RFX6*, *KCNJ11* (2). We excluded *CEL*-MODY from this analysis as it is not possible to call the single base deletions that cause *CEL*-MODY from whole exome sequencing data as they occur in the gene's variable number tandem repeat region (24, 25). We annotated these variants using Ensembl's Variant Effect Predictor (26). We classified as pathogenic or likely pathogenic according to ACMG/AMP guidelines (27, 28). To minimize the inclusion of false-positive variants, we only included protein-truncating variants (PTVs) in the study if they were classified as high confidence by LOFTEE (29). To further limit the inclusion of very low-penetrance variants, we only included pathogenic missense variants if they were ultra-rare in the population (maximum allele count of 2 in gnomAD v2.1.1, MAF <1.4 × 10^−5^). We manually reviewed sequence read data for all pathogenic protein-truncating variants using the Integrative Genomics Viewer to exclude false-positive variants (30). The variants included in the analysis are listed in Table S2 (18). We also included individuals with *HNF1B*-MODY caused by deletions in the 17q12 region, as nearly half of the *HNF1B*-MODY cases are due to this deletion (31). We used genotyping array data for this analysis, as described in previous work by Cannon et al (31).

### Statistical Analysis

We statistically analyzed proportions/counts between categories and groups using the Fisher exact test. To calculate 95% CIs for proportions, we applied the exact binomial confidence interval Clopper–Pearson method. We used Kaplan-Meier survival analysis to estimate age-dependent penetrance of diabetes and all-cause mortality. To compare diabetes penetrance and all-cause mortality between groups, we performed the log-rank test. We computed hazard ratios for diabetes and all-cause mortality using Cox proportional hazard models, both with and without adjustment for covariates. We assessed the impact of age on HbA1c levels through linear regression. Welch 2-sample t-tests were used to compare HbA1c and blood glucose levels between *GCK* heterozygotes and individuals without pathogenic GCK variant. We evaluated heterogeneity in prevalence by ancestry using Cochran's Q test using STATA package META. We conducted all analyses using STATA 18.0 and R version 4.2.2 with RStudio.

## Results

### At Least 1 in 1052 Individuals in the UK Biobank Carries a Pathogenic MODY Variant, With *GCK* Variants Being the Most Common Seen in 1 in 2787 Individuals

**Figure 1.**
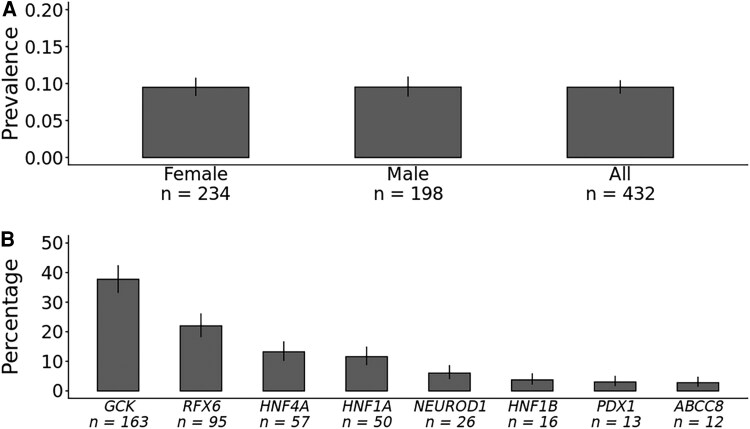
Prevalence of pathogenic variants in 10 MODY genes in a population cohort. A, The graph shows the prevalence (%) of pathogenic variants associated with 10 MODY genes across 454 275 individuals from the UK Biobank (n = 432 carriers), with data stratified by sex (female, 234/246 437 and male 198/207 838). B, The distribution of pathogenic variants among the 432 carriers from the UK Biobank, categorized by genetic etiology. Error bars indicate 95% CI.

Our analysis of variants in 10 MODY genes across 454 275 UK Biobank participants identified 432 individuals carrying pathogenic MODY variants. This represents a prevalence of 0.095% (95% CI 0.086-0.1, 1:1052) (Fig. 1A). Unlike clinically selected cohorts, male and female individuals with diabetes showed similar prevalence rates (0.095% vs 0.095%, *P* = 1). Pathogenic variants in *GCK* were most prevalent, comprising 37.7% (163/432) of cases, followed by pathogenic variants in *RFX6* at 22% (95/432) (Fig. 1B). Overall pathogenic variants in HNF1A/4A/1B transcription factor genes (*HNF1A*/*HNF4A*/*HNF1B*) were responsible for 28.5% cases, whereas pathogenic variants in other rare MODY genes were responsible for 33.8% cases. The population prevalence of *GCK*-MODY was 1:2787 (0.036%, 95% CI 0.031-0.042%) and this rose to 1:1013 (0.099%, 95% CI 0.084-0.12%) in people with prediabetes or diabetes. The population prevalence of pathogenic HNF1A/4A/1B MODY variants was 0.027% (123/454 275, 1 in 3693) and pathogenic variants in other rare MODY genes was 0.032% (146/454 275, 1 in 3111). Among individuals with diabetes at recruitment, 0.63% carried pathogenic MODY variants (95% CI 0.54-0.73%, 181/28 795, 1 in 159). The prevalence of MODY was 1 in 68 for those diagnosed before age 40 (1.48%, 95% CI 1.06-2.01%, 40/2706), and 1 in 141 to those diagnosed before age 60 years (0.71%, 95% CI 0.6-0.84%, 136/19 187). The distribution of genetic etiologies in all diabetes, diabetes diagnosed at age < 40 years, and those diagnosed < 60 years are provided in Table S3 (18). The prevalence of pathogenic MODY variants remained similar across European (0.096%), South Asian (0.12%), and other (0.091%) ancestries, although individuals of African ancestry showed significantly lower prevalence (0.014%) (*P*_heterogeneity_ 2.1 × 10^−7^) (Table S4) (18).

### Pathogenic *GCK* Variants Show High Penetrance in a Population Cohort

**Figure 2.**
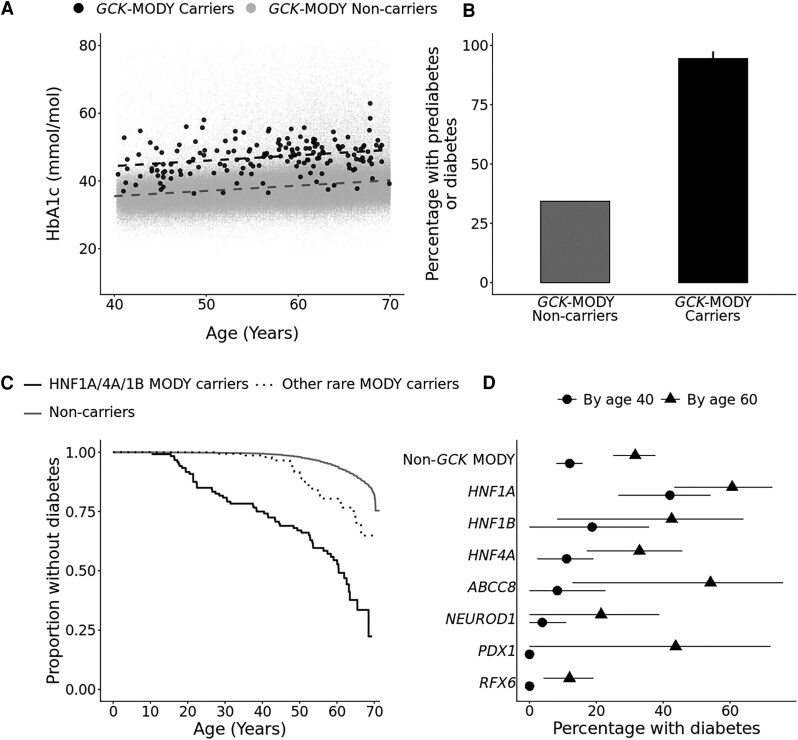
Penetrance of pathogenic MODY variants in the population cohort. A, Scatter plot illustrating the relationship between HbA1c (mmol/mol) and age at recruitment for individuals with *GCK* pathogenic variant (n = 161) and individuals without *GCK* pathogenic variant (n = 432 107). Dashed lines represent linear trends for each group. B, Bar chart displaying the percentage of individuals with prediabetes or diabetes in individuals with and without pathogenic *GCK* variant, with error bars indicating 95% CI. C, Kaplan-Meier analysis of diabetes onset in UK Biobank participants, stratified by pathogenic MODY variant carrier status (HNF1A/4A/1B MODY carriers [genes = *HNF1A*, *HNF4A*, *HNF1B*], n = 120; other rare MODY carriers [genes = *INS*, *PDX1*, *NEUROD1*, *RFX6*, *KCNJ11*, *ABCC8*], n = 146; Non-carriers n = 452 916). D, The proportion of pathogenic MODY variant carriers developing diabetes by age 40 (circle) and age 60 (triangle), with 95% CI.

Pathogenic variants in *GCK* have been shown to cause mild lifelong fasting hyperglycemia in clinically ascertained cases (32). We found that individuals with pathogenic *GCK* variants showed, on average, 8.8 mmol/mol higher HbA1c levels compared with individuals without pathogenic variants in *GCK* in the UK Biobank (47.1 ± SD 4.9, n = 161 vs 38.4 ± 6.4 mmol/mol, n = 432 107, *P* = 7.61×10^−69^) (Fig. 2A). Similarly, their glucose after at least 5 hours of fasting was 1.28 mmol/L higher compared with individuals without pathogenic variants in *GCK* (6.34 ± 0.97, n = 40 vs 5.06 ± 1.04 mmol/L, n = 93 307, *P* = 5.4×10^−15^). The differences between the groups were maintained after adjusting for age, sex, body mass index (BMI), parent diabetes status, and genetic ancestry principal components (Table S5) (18).

The individuals with a pathogenic *GCK* variant showed an age-dependent increase in HbA1c (beta 0.17, 95% CI 0.17-0.17, *P* = 7.17 × 10^−4^), which was similar to individuals without pathogenic variants in GCK (*P*_interaction_ .76). The prevalence of prediabetes or diabetes in individuals with pathogenic *GCK* variant was 94.5% (95% CI 89.8%-97.4%) compared with 34.3% (95% CI 34.2%-34.4%) in individuals without a pathogenic GCK variant (*P* = 1.21×10^−59^) (Fig. 2B).

### Non-*GCK* Pathogenic MODY Variants Demonstrate Variable Age-Dependent Diabetes Penetrance Across Genetic Etiologies

We next assessed the age-dependent risk of diabetes in individuals with pathogenic variants in non-*GCK*-MODY genes within our population cohort. By age 40 years, 12% (95% CI 8%-15.9%) of non-*GCK* pathogenic MODY variant carriers had developed diabetes, increasing to 31.6% (95% CI 25%-37.7%) by age 60 years. However, the diabetes penetrance varied substantially across genetic etiologies (Fig. 2C and 2D). *HNF1A* pathogenic variants emerged as the most penetrant cause of diabetes, with 42% (95% CI 26.6%-54.2%) of carriers developing diabetes by age 40 years, rising to 60.6% (95% CI 43.3%-72.7%) by 60 years. In contrast, *RFX6*, *PDX1*, and *NEUROD1* pathogenic variants showed the lowest penetrance. No *RFX6* or *PDX1* carriers had diabetes by age 40 years while only 3.8% of *NEUROD1* carriers had diabetes. By 60 years, 12% of *RFX6* carriers, 43.8% of *PDX1* carriers, and 21.4% of *NEUROD1* carriers had diabetes (Fig. 2D). Together, 25% (95% CI 16.8%-32.4%) of individuals with pathogenic HNF1A/4A/1B variants developed diabetes by age 40 and 45.5% (95% CI 34.9%-54.4%) by age 60, compared with 1.4% (95% CI: 0%-3.2%) and 19.6% (95% CI 11.7%-26.8%) of individuals with pathogenic variant in other rare MODY genes respectively (Fig. 2C). The analysis of the relative risk by Cox proportional hazard models showed consistent results where risk of diabetes was varied by the genetic etiology (hazard ratio [HR] 6.84, 95% CI 5.54-8.46, *P* = 6.83 × 10^−71^ all variants; HR 12.1, 95% CI 9.38-15.6, *P* = 1.36 × 10^−81^ for HNF1A/4A/1B variants; and HR 3.5, 95% CI 2.4-5.1, *P* = 7.87 × 10^−11^ for other rare MODY gene variants). The results were consistent with or without adjusting for sex, parental diabetes status, age at recruitment, BMI, and genetic ancestry principal components (Table S6) (18).

### Type 2 Diabetes Polygenic Score and Parental Diabetes Status Affect the Penetrance of Pathogenic MODY Variants

Our genotype-first approach allowed us to assess the true impact of environmental and polygenic factors on age-dependent diabetes penetrance. We found that sex, BMI, and type 1 diabetes genetic risk had no effect on diabetes penetrance in carriers of pathogenic non-*GCK*-MODY variants (Table 1). However, parental history of diabetes increased the diabetes risk ∼2-fold (HR 2.54, 95% CI 1.56-4.14), and each SD increase in type 2 diabetes polygenic risk score increased the risk by 1.52-fold (95% CI 1.14-2.03, *P* = 3.9 × 10^−3^) (Table 1). These results were directionally consistent across genetic etiologies but were limited by small numbers in each group (Table S7) (18).

**Table 1.** Factors influencing diabetes penetrance in pathogenic non-*GCK*-MODY pathogenic variant carriers

Factor	Hazard ratio*^a^* (95% CI)	*P* value	Adjusted hazard ratio*^b^* (95% CI)	*P* value
Sex, male	1.12 (0.72-1.76)	.61	1.21 (0.74-1.97)	.45
BMI*^c^*	1.1 (0.87-1.38)	.43	1.15 (0.91-1.46)	.25
Parent with diabetes	2.54 (1.56-4.14)	1.83×10^−4^	2.26 (1.35-3.81)	2×10^−3^
T1DGRS*^c^*	0.96 (0.76-1.21)	.72	0.94 (0.73-1.2)	.61
T2DPRS*^c^*	1.52 (1.14-2.03)	3.94×10^−3^	1.46 (1.08-1.96)	.013

Abbreviations: BMI, body mass index; MODY, maturity-onset diabetes of the young; T1DGRS, type 1 diabetes genetic risk score; T2DPRS, type 2 diabetes polygenic risk score.

^*a*^Cox proportional hazards model, adjusted for ancestry principal components and genetic etiology.

^*b*^Hazard ratio adjusted for ancestry principal components, genetic etiology, age at recruitment, sex, BMI, parental diabetes status, type 1 and type 2 diabetes polygenic risk scores (T1DGRS and T2DPRS).

^*c*^Continuous variables are standardized.

### Pathogenic MODY Variants Carriers Do Not Show Excess All-Cause Mortality

**Figure 3.**
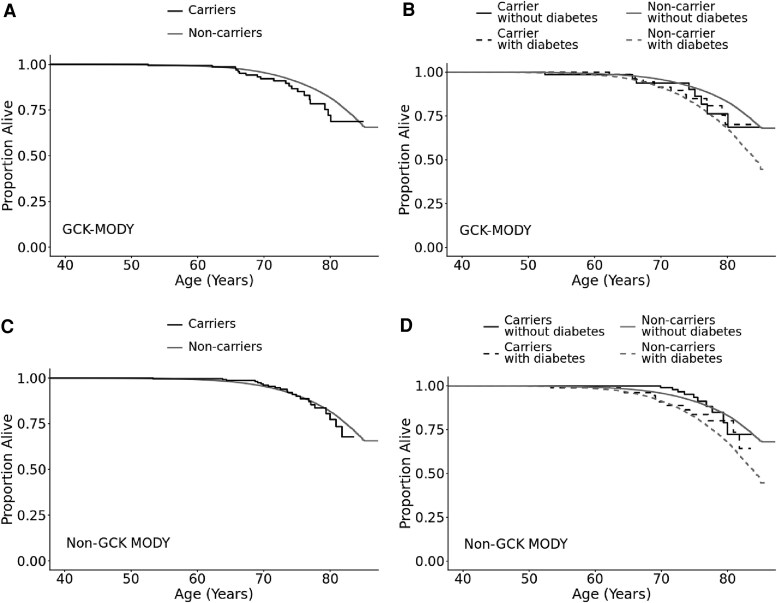
All-cause mortality in pathogenic MODY variant carriers in a population cohort. Kaplan-Meier analysis of all-cause mortality in participant from the UK Biobank split by: A, *GCK* pathogenic variant carrier status (pathogenic *GCK*-MODY carriers n = 163; non-carriers n = 454 112) (log-rank *P* = .09). B, *GCK* pathogenic variant carrier status and diabetes status (pathogenic *GCK*-MODY carriers with diabetes n = 92; pathogenic *GCK*-MODY carriers without diabetes n = 71; non-carriers with diabetes n = 28 703; non-carriers without diabetes n = 425 409) (pathogenic *GCK*-MODY carriers with diabetes vs non-carriers with diabetes, log-rank *P* = .34) (pathogenic *GCK*-MODY carriers without diabetes vs non-carriers without diabetes, log-rank *P* = .2). C, Non-*GCK* pathogenic variant carrier status (pathogenic non-*GCK*-MODY carriers n = 269, non-carriers n = 454 006) (log-rank *P* = .89). D, Non-*GCK* pathogenic variant carrier status and diabetes status (pathogenic non-*GCK*-MODY carriers with diabetes n = 89; pathogenic non-*GCK*-MODY carriers without diabetes n = 180; non-carriers with diabetes n = 28 706; non-carriers without diabetes n = 425 300) (non-*GCK*-MODY carriers with diabetes vs non-carriers with diabetes, log-rank *P* = .38) (non-*GCK*-MODY carriers without diabetes vs non-carriers without diabetes, log-rank *P* = .51).

Finally, we assessed whether the individuals with pathogenic variants in MODY genes were at higher risk of all-cause mortality compared to individuals without pathogenic variants in MODY genes. Despite higher HbA1c levels, over a mean follow-up duration of 13.4 years at mean age of 70.4 years, individuals with pathogenic *GCK* variants did not show excess all-cause mortality compared to individuals without pathogenic GCK variants in the UK Biobank (log-rank *P* = .09) (Fig. 3B). The results were similar irrespective of the diabetes status (log-rank *P* = .34 for individuals with diabetes and pathogenic variants in *GCK* vs individuals with diabetes without pathogenic variants in *GCK*; log-rank *P* = .2 for individuals without diabetes and pathogenic variants in *GCK* vs individuals without diabetes and without pathogenic variants in *GCK*). The all-cause mortality for *GCK* pathogenic variant carriers remained similar, regardless of whether they had diabetes or had HbA1c levels above or below the median of the GCK groups (Table S8 and Fig. S1) (18).

We also did not observe excess all-cause mortality for individuals with pathogenic variants in non-*GCK*-MODY genes compared with individuals without pathogenic variants (log-rank *P* = .89) (Fig. 3A). The results were also similar irrespective of the diabetes status (all log-rank *P* > .1) (Fig. 3B). The results were also consistent across all MODY subtypes, both individually and combined (*P* > .05) and after adjusting for sex, smoking status, diabetes status, age at recruitment, and HbA1c (Table S8) (18).

## Discussion

In this large population study using a genotype-first approach, we show that MODY is prevalent in the population (1:1052) and accounts for 1.48% of diabetes under 40 years. The penetrance is much lower than previously estimated and varies substantially by genetic etiology, family history, and polygenic type 2 diabetes background. We also show that MODY does not cause excess all-cause mortality, even in individuals with *GCK*-MODY who have lifelong excess glucose burden.

Our genotype-first approach with a large sample size provides novel insights over phenotype-first studies. We report a prevalence of *GCK*-MODY of 1:2787 (0.036%). This is lower than the currently widely adopted estimate of ∼1:1000 by Chakera et al (33). However, this previous estimate is based on a small study of 247 pregnant women with glucose >5.1 with only 4 cases of *GCK*-MODY; in contrast we had 163 cases of *GCK*-MODY with sample size of 454 275, potentially providing a more accurate prevalence estimate. The overall MODY prevalence in our population cohort was 1:1052 (0.095%), nearly 3 times higher than a previous estimate of ∼1:3521 (284 cases/million) in the UK (34). This difference likely reflects phenotype-based estimation of this study.

We also found that MODY accounted for 1.48% of diabetes cases diagnosed before age 40. Shields et al (35) reported a MODY prevalence of 3.6% (51/1407) in individuals diagnosed younger than 30 years in a large, population-based diabetes study in south-west England. In the equivalent age group in UK Biobank, we observed a prevalence of 2.1% (28/1312). The mean age of diabetes diagnosis was higher in UK Biobank compared to Shields et al (18.2 vs 13 years), highlighting the underrepresentation of young-onset diabetes in UK Biobank. Similarly, previous studies have shown that approximately 2.5% to 5% of pediatric diabetes cases are MODY (36, 37), whereas our observed prevalence in this age group was 1.3%. These data together suggest that UK Biobank is underrepresented for young-onset diabetes, and our estimates should be interpreted as minimum prevalence figures, with the true prevalence expected to be higher in the general population. This difference is potentially due to healthy participant bias in the UK Biobank, leading to likely lower representation of young-onset diseases, and the inclusion of m.3243A>G, and lipodystrophy genes in previous studies (38, 39). A significantly lower prevalence of pathogenic MODY variants in individuals of African ancestry has been reported with other monogenic diseases (40, 41). This likely reflects the lack of sufficient available genetic evidence to classify novel or previously observed variants as pathogenic in this ancestry. Finally, the lack of female excess in our study, despite higher female participation in UK Biobank, strongly suggests that female overrepresentation in clinically ascertained cohorts results from an ascertainment bias.

Carriers of pathogenic *GCK* variants do not show excess all-cause mortality. Previous clinically ascertained cases demonstrated that *GCK*-MODY has high fasting glucose that increases with age (32). Here, using a large number of clinically unselected cases (163 individuals: mean age 56.8 years), we observed similar findings. We also found that despite an average HbA1c of 47.1 mmol/mol, these individuals did not show excess all-cause mortality. These findings strongly support current guidelines, which suggest discharging individuals with *GCK*-MODY from follow-up. Prediabetes in the general population is associated with an increased risk of all-cause mortality (42). In contrast, individuals with GCK-MODY, who have lifelong mild fasting hyperglycemia, did not show increased all-cause mortality in our cohort. This indicates that isolated impaired fasting glucose, even when present from birth, is not sufficient to increase mortality risk. The excess mortality seen in population-based studies of prediabetes is therefore likely explained by coexisting metabolic abnormalities such as dyslipidemia, obesity, hypertension, and chronic kidney disease, rather than mild hyperglycemia alone.

We did not observe excess mortality with any non-*GCK*-MODY subtype. This is particularly of note for *HNF1A*, where our findings contrast with previous studies. Steele et al showed that clinically ascertained families with the *HNF1A* p.Pro291fsinsC variant had increased overall and cardiovascular mortality (12). Similar observation of all-cause mortality reported in Icelandic population in clinically unselected population for this particular variant (43). The discrepancy in results may be due to multiple factors. The healthy participant bias and minimum recruitment age of 40 years in the UK Biobank means young-onset cases who died before 40 or severe cases would not be in the UK Biobank. The substantially improved cardiovascular preventative therapy and lower cardiovascular mortality over time in the UK general population may have lowered mortality in our cases (44, 45), and finally, difficulties in accurately calling p.Pro291fsinsC variant from exome sequencing meant our study may have underrepresented this variant (only 16/60 individuals were confirmed to have the variant on manual check using Integrative Genomics Viewer), which could have reduced the overall effect of *HNF1A* variants on all-cause mortality (10, 11).

Our genotype-first approach shows that non-*GCK*-MODY variants have variable penetrance and allowed us a unique insight into the heterogeneity of diabetes risk within the known MODY genes. We demonstrated that MODY variants increase diabetes risk as expected, but the absolute risk is much lower than clinically ascertained cases and varies substantially by genetic etiology. We observed that *HNF1A* was the most penetrant with 60.6% developing diabetes by age 60 years, whereas *ABCC8*, *HNF1B*, and *HNF4A* genes showed moderate penetrance (32.9%-54.2% by 60 years) and *NEUROD1*, *PDX1*, and *RFX6* genes showed the lowest penetrance (12-43.8% by 60 years). This lower penetrance in clinically unselected cohorts has been observed previously for *HNF1A* and *HNF4A* (10, 14). In this study, we extend this observation to all MODY genes. The lower penetrance in clinically unselected cohorts likely represents the lower bound of true penetrance, whereas clinical cohorts show the upper bound. The wide difference in penetrance between phenotype-first and genotype-first studies is not limited to diabetes but has been reported in other monogenic disorders (46-49). This wide range in penetrance suggests multiple modifiers are likely at play. In our limited sample size, we demonstrated that parental diabetes and polygenic background of type 2 diabetes independently affect penetrance likely due to overlapping pathophysiological pathway, an observation previously reported in clinically ascertained cases (50, 51).The lack of impact of T1DGRS and BMI on MODY penetrance suggests that potential modifiers need to share a pathophysiological pathway with MODY genetic causes, which primarily involve beta cell dysfunction rather than autoimmune or adipocyte function. Further studies with larger numbers are needed to assess which pathways of type 2 diabetes genetic risk underlie this effect.

Our study has several limitations. We used a large population cohort in our study, but the UK Biobank has shown to have a healthy participant bias partly due to a minimum recruitment age of 40 years (38). This has likely caused an underestimation of penetrance, prevalence, and mortality. Despite using the largest cohort to date, we were still limited by the number of pathogenic carriers in some genes. Our cohort predominantly contains individuals of European ancestry, which may limit the generalizability of our findings to other populations.

In summary, we showed that 1 in 1052 individuals in the population have a pathogenic MODY variant and that MODY accounts for 1.48% of diabetes cases under 40 years, with no impact on all-cause mortality and substantially lower penetrance than previously thought.

## Data Availability

The UK Biobank dataset is available to researchers from https://biobank.ctsu.ox.ac.uk. The variants used in this study are available in the manuscript. Original data generated and analyzed during this study are included in this published article or in the data repositories.

## Abbreviations

BMI: body mass index
MODY: maturity-onset diabetes of the young
T1DGRS: type 1 diabetes genetic risk score
T2DPRS: type 2 diabetes polygenic risk score
